# Hyaluronan Inhibition as a Therapeutic Target for Diabetic Kidney Disease: What Is Next?

**DOI:** 10.34067/KID.0000000000000126

**Published:** 2023-04-14

**Authors:** Loay Salman, Laisel Martinez, Geovani Faddoul, Christina Manning, Karim Ali, Maya Salman, Roberto Vazquez-Padron

**Affiliations:** 1 Division of Nephrology and Hypertension, Department of Medicine, Albany Med Health System, Albany, New York; 2 Division of Vascular Surgery, DeWitt Daughtry Family Department of Surgery, University of Miami Leonard M. Miller School of Medicine, Miami, Florida; 3 Faculty of Medicine, Damascus University, Damascus, Syria

**Keywords:** diabetic kidney disease (DKD), CKD, 4-methylumbelliferone (4-MU), hyaluronan (HA), hymecromone, 4-methylumbelliferyl glucuronide (4-MUG), 4-methylumbelliferyl sulfate (4-MUS)

## Abstract

Diabetic kidney disease (DKD) is the leading cause of CKD and ESKD in the United States and worldwide. Pharmacotherapy and lifestyle modifications for glycemia, dyslipidemia, and BP control have shown success in slowing the progression of DKD. Traditional treatments, such as angiotensin-converting enzyme inhibitors or angiotensin receptor blockers and more recently the use of sodium-glucose cotransporter 2 inhibitors, nonsteroidal selective mineralocorticoid receptor antagonists, such as finerenone, and glucagon-like peptide 1 receptor agonists, have led to added benefits on various outcomes. However, significant residual risk for DKD progression remains despite the current standard-of-care approaches. Arteriolar hyalinosis (AH) is among the key findings seen on kidney biopsies of patients with DKD. It results from the excessive accumulation of hyaluronan (HA) in the arterioles. AH has not been targeted specifically by any of the therapeutic methods currently being used. We discuss in this manuscript the potential use of a selective therapy targeting AH and the increased total renal HA deposits using a HA synthesis inhibitor in DKD.

## Introduction

Diabetic kidney disease (DKD) is the leading cause of CKD and ESKD in the United States and worldwide.^1^ An estimated 37 million people in the United States have diabetes mellitus (DM) and more than a third of the population, approximately 96 million adults, is prediabetic.^1^ On average, one in three adults with DM develop DKD.^1^ Thirty-nine percent of patients with ESKD status in the United States are attributable to DKD.^1^ DKD is a serious complication of DM that contributes significantly to patients' morbidity and mortality, along with the addition of a monumental financial burden to our health care system.

**Figure 1:**
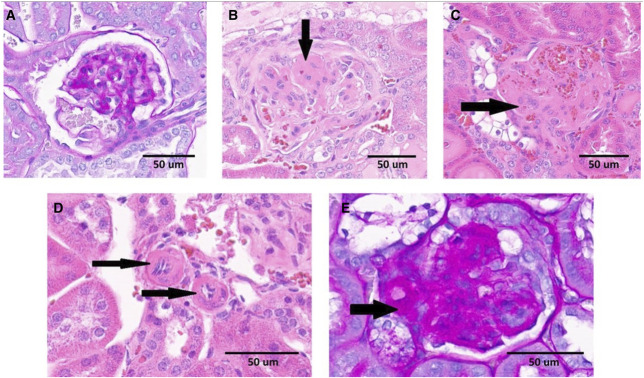
**Kidney histopathology in diabetic mice.** Examples of (A) a normal glomerulus; (B) glomerulus with mesangial expansion, segmental glomerulosclerosis, and a Kimmelstiel-Wilson nodule (arrow); (C) glomerular mesangiolysis; and (D and E) AH. (A and E) Correspond to PAS staining. (B–D) Stained with hematoxylin and eosin. (A) Obtained from 4-MU–treated mice. (B–E) Originated from diabetic animals treated with control diet. Adapted from ref. 3 with permission. PAS, periodic acid–Schiff.

The initial pathologic change in DKD is the thickening of the glomerular basement membrane.^2^ Subsequent typical and important pathologic changes seen are diffuse or nodular mesangial expansion (such as Kimmelstiel-Wilson nodules), podocyte injury, arteriolar hyalinosis (AH) (Figure 1, D and E), and arteriosclerosis of larger vessels.^4^ Glomerular sclerosis and tubulointerstitial fibrosis are considered late manifestations of DKD leading to advanced CKD and ESKD.^5^

Several potential overlapping pathways contribute to the complex pathogenesis of DKD. Hyperglycemia, hyperinsulinemia, and insulin resistance play important roles in inciting these pathways. Glomerular hyperfiltration is a well-known complication of DM caused by increased renal plasma flow, increased filtration fraction, and renal hypertrophy because of the overall diabetic milieu.^6^ Impaired renal autoregulation further exacerbates glomerular hyperfiltration.^7^ Inflammation plays an important role in the pathogenesis of DKD which transpires because of activation of proinflammatory and profibrotic pathways and gene expression as a result of the production of advanced glycation end-products and reactive oxygen species because of hyperglycemia.^8,9^ Macrophages play a key role in inflammation in DKD, and the magnitude of renal infiltration correlates with disease progression and outcome.^10^ In addition, increased activity of protein kinase C, reduced production of endothelial nitric oxide synthase (eNOS), increased levels of endothelin 1, increased vascular endothelial growth factor, and increased transforming growth factor-*β* play an important role in endothelial instability, mesangial cell hypertrophy, and mesangial expansion in DKD.^11,12^ VEGF is believed to be the reason leading to increased vascular proliferation and endothelial permeability in DKD.^13^

There have been important advances in the treatment of DKD over the past few decades. Lifestyle modifications to achieve glycemic control and BP control to target, along with lowering plasma lipids, have shown success in slowing the progression of DKD.^14–25^ Mainstay antihypertensive regimens with angiotensin-converting enzyme inhibitors or angiotensin receptor blockers are now combined with more recent drug classes for better risk reduction. Specifically, sodium-glucose cotransporter 2 inhibitors, nonsteroidal selective mineralocorticoid receptor antagonists, such as finerenone, and glucagon-like peptide 1 receptor agonists have demonstrated significant cardiorenal benefits in patients with DKD.^26–29^ However, each line of treatment has its own limitations, adverse reactions, and contraindications, as well as added associated costs which limit their use.^28,30–38^ In addition, there is residual risk of DKD leading to progression to ESKD despite all abovementioned treatment regimens. These challenges highlight the need for additional agents with novel therapeutic targets. It is important to mention that certain common pathologic findings in DKD, such as increased total renal hyaluronan (HA) content and AH, are not specifically targeted by any of the abovementioned therapeutic agents.

## HA and Kidney Disease

HA, a polymer of hyaluronic acid, accumulates in the kidneys of patients with DM leading to specific pathologic findings, such as AH.^5^ It is generally believed that high molecular weight HA (2×10^5^ to 2×10^6^ Da) is present in the steady state and has anti-inflammatory properties, whereas the low molecular weight (LMW) HA (<100 kDa) predominates at sites of active inflammation.^39^ The main receptors for HA are CD44 and receptor for hyaluronic acid–mediated motility.^39^ There is increased overall accumulation of HA in kidneys among patients with DKD. AH results from HA specifically accumulating in glomerular afferent and efferent arterioles.^40,41^ This increased renal HA accumulation has been linked to worsened proteinuria, faster loss of kidney function, and increased cardiovascular events.^3,41,42^ Afferent and efferent AH is one of the key pathologic findings in DKD.^5^ There have been various pathologic classifications of AH on the basis of the number of arterioles affected, such as the criteria proposed by Tervaert *et al.*,^43^ or the wall thickness and percentage of luminal area occlusions in the most severely affected arteriole^44^ or the number of arterioles affected and the circumferences of the arterioles involved, as in the Banff lesion score.^45^ In a study of 377 patients with type 2 DM and biopsy-proven DKD and with a median follow-up of 5.9 years, Morimoto *et al.* discovered that AH, and not intimal thickening of larger arteries, was strongly associated with increased cardiovascular events, ESKD incidence, and proteinuria in people with diabetic nephropathy.^42^ Another study by Oguchi *et al.* evaluating 248 kidney transplants with 381 biopsy specimens showed that vasa recta hyalinosis (VRH) in the medulla of renal allografts is associated with worse graft survival rate.^46^ One potential pathophysiologic outcome of AH is the reduction of the interstitial capillary bed and the glomerular blood flow, both leading to glomerular sclerosis. Another study of 109 patients with CKD who underwent renal biopsy shows that AH may potentiate susceptibility to BP-related glomerular damage because of dysregulated afferent and efferent arteriolar resistance.^47^ Therefore, one of the main effects of AH on the glomerulus is through impeding autoregulation. In a study of 143 glomerular-arteriolar pairs, it was shown that hyaline arteriolosclerosis may lead to the loss of autoregulation, possibly due to thinning and degeneration of the underlying smooth muscle.^48^ This effect on autoregulation is not a new finding and has been known for a relatively long time.^49,50^ Interestingly, in diabetic patients, AH exists in other organs, such as the brain, heart, and other organs, and has shown to lead to impaired autoregulation within the organ leading to certain clinical manifestations^51^ and organ-specific complications.^48^

In summary, afferent and efferent AH are associated with increased ESKD incidence, proteinuria, increased cardiovascular events, worse renal graft survival, and worse glomerular disease outcomes.^42,46^ In fact, one of the key ways AH leads to a worse outcome in DKD is through impairment of renal autoregulation resulting in glomerular sclerosis by subjecting the renal glomerulus to the effects of low or high systemic BP.^47^

## HA Synthesis Inhibitors

**Figure 2:**
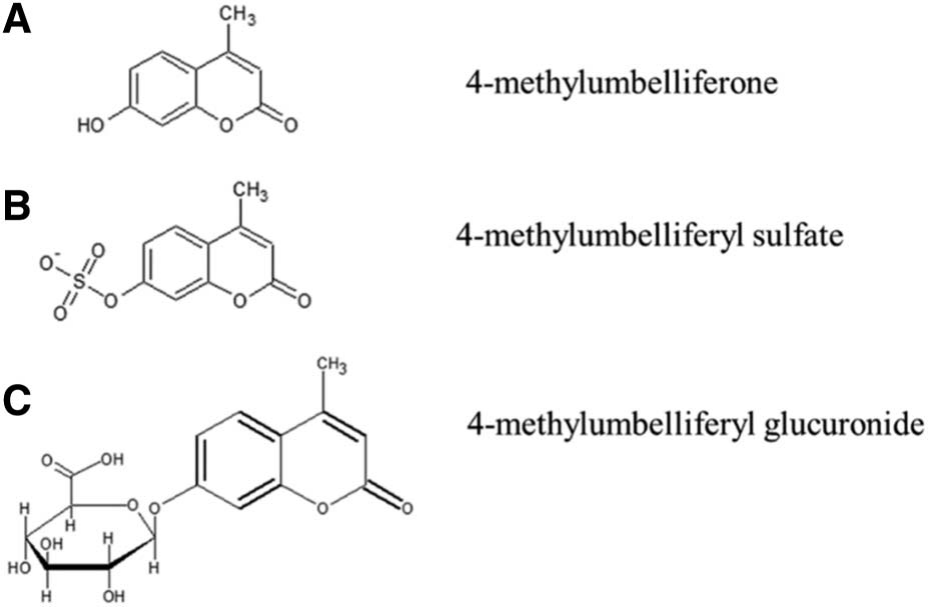
**Molecular structure of 4-MU and its metabolites.** Adapted from ref. 39 with permission.

**Figure 3:**
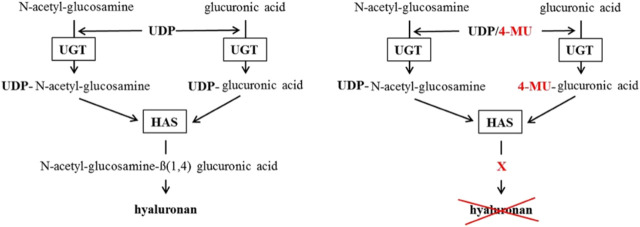
**Postulated** 4-MU **mechanism of HA synthesis inhibition.** The left scheme shows the normal way HA gets synthesized. The right scheme shows how 4-MU binds to GlcUA instead of UDP so the HAS cannot build HA. HAS, HA-synthase. Adapted from ref. 39 with permission.

4-methylumbelliferone (4-MU) (Figure 2) is a derivative of coumarin, with the molecular formula C_10_H_8_O_3_, a molecular weight of 176.2 kDa, a chemical abstract service number of 90-33-5, a pKa of 7.79, and a melting point of 194–195°C.^39^ 4-MU inhibits HA synthesis by binding through its hydroxyl group at position 4 to glucuronic acid (GlcUA) *via* the UDP-glucuronosyltransferase (UGT), thus acting as a competitive substrate for UGT which leads to reduction in UDP-GlcUA in the cytosol and, therefore, less HA synthesis (Figure 3).^39^ Another mechanism by which 4-MU inhibits HA synthesis is by downregulating the expression of HA synthase 2 and/or 3 (HAS2 and/or HAS3).^39,52^ 4-MU is metabolized extensively in the liver with <1% excreted unchanged in the urine.^39^ 4-MU is metabolized to either 4-methylumbelliferyl glucuronide (4-MUG) (over 90%) or 4-methylumbelliferyl sulfate (Figure 2).^39^

Nagy *et al.* has recently shown that 4-MUG directly and indirectly inhibits HA synthesis independently of 4-MU.^53^ Importantly, they also discovered that mice fed either 4-MUG or 4-MU had equivalent 4-MU:4-MUG ratios in the serum, liver, and pancreas, indicating that 4-MU and 4-MUG reach equilibrium in these tissues.^53^

## HA Synthesis Inhibitors Limitations

4-MU has potential unfavorable outcomes. In one study, 4-MU led to a reduction in the ability of the kidney to respond appropriately on acute hydration given to 4-MU–treated rats.^54^ This is speculated to be due to the role HA plays as a tubular fluid handling modulator by changing the physicochemical properties of the interstitial space. However, this was not a model of DKD; therefore, treatment likely targeted normal HA levels but not the abnormal accumulation seen in diabetic patients. Another potential unfavorable outcome is that 4-MU was associated with worse atherosclerosis in Atherosclerosis-prone apolipoprotein E-deficient mice fed a high-fat diet.^55^ This finding is speculated to be due to endothelial glycocalyx damage facilitating leukocyte adhesion and inflammation.^55^ These results may also be model-specific as vessels in rodents are known to have a different content of glycosaminoglycan (including lower HA) when compared with human and other atheroprone species vessels.^56^ In fact, overproduction of HA in the aorta of HAS2 transgenic Atherosclerosis-prone apolipoprotein E-deficient mice resulted in accelerated atherosclerosis.^57^ Despite differences between species, both of these potential unfavorable outcomes should be appropriately monitored when conducting clinical studies.

## HA Inhibitors in DKD

We have conducted a study to assay the effects of the HA synthesis inhibitor, 4-MU, on the progression of DKD.^3^ We used the eNOS^−/−^ C57BLKS/J^db^ mouse model for these experiments. The double homozygous mouse develops type 2 DM and becomes moderately hypertensive. Double homozygous mice were separated at age 9 weeks into two matched groups. Treated animals were fed a 4-MU–containing diet while control animals were fed a regular diet. Both groups were compared with their heterozygous siblings, which were fed a regular diet. Although treatment and control groups had similarly elevated albumin-to-creatinine ratios (ACRs) at the beginning of the experiment, 9 weeks later the 4-MU–treated group had significantly lower ACR than their diabetic controls. Similarly, GFR was not different between the groups at the beginning of the experiment, although both groups clearly had hyperfiltration. At the end of the experiment (week 18), the 4-MU–treated group's GFR was similar to baseline (*P* = 0.7) while the control group's GFR was significantly lower than the 4-MU–treated group's GFR (*P* = 0.042). It is important to mention that GFR in these experiments was measured by using FITC-labeled inulin method. The results were comparable when cystatin C was used for analysis.

**Figure 4:**
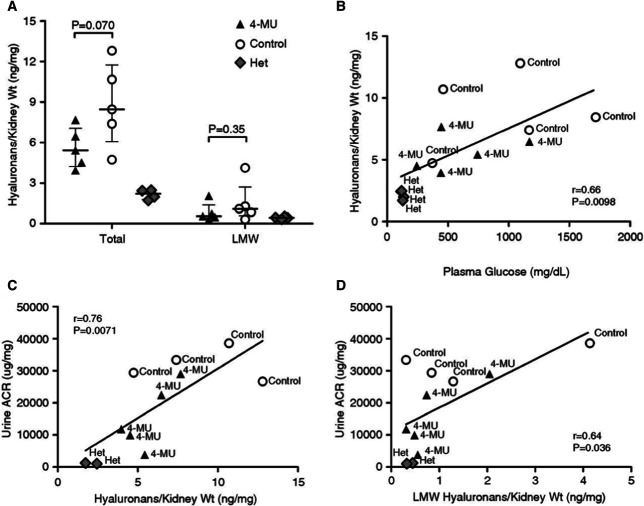
**Kidney HAs and correlation with plasma glucose and renal function tests.** (A) Total and LMW HAs in kidneys from 4-MU–treated (*n*=5), control (*n*=5), and heterozygous nondiabetic littermate mice (*n*=4) at week 18. Values are expressed as HA content per milligram of kidney wet weight. Error bars indicate the median and IQR. Groups were compared using a *t* test or Mann–Whitney test. (B) Correlation between nonfasting plasma glucose and total kidney HA content at week 18. (C and D) Correlations between total HA (C) or LMW HA content (D) in kidneys and urine ACR at week 17. The *y* axis values were not available for one control mouse and two heterozygous animals in these analyses. Adapted from ref. 3 with permission. IQR, interquartile range.

Interestingly and unexpectedly, the 4-MU–treated group had significantly lower average fasting plasma glucose at the end of the experiment when compared with the control group, although both groups remained diabetic, and their average fasting plasma glucose levels increased over time during the study. Kidney morphology analysis showed that kidney weight was 42% higher in the control diabetic animals (64% when normalized to body weight) when compared with 4-MU–treated animals at the end of the experiment. Kidney weights of the 4-MU–treated group were similar to the nondiabetic heterozygous group (*P* = 0.76). Histopathology analysis showed significantly higher glomerular injury index and mesangial expansion in the diabetic control group when compared with the 4-MU–treated group (Table 1 taken from ref. 3 with permission). Both diabetic groups had higher HA kidney content when compared with their nondiabetic siblings, highlighting the pathologic HA deposits as a complication of DM. The 4-MU–treated group showed a trend of 36% reduction in total kidney content of HA when compared with the diabetic controls (*P* = 0.07). Interestingly, the plasma glucose level strongly correlated with the total kidney HA content. Both total HA and LMW HA levels in kidneys strongly correlated with urine ACR (Figure 4 taken from ref. 3 with permission). Importantly, there were no signs of severe AH, tubulitis, or nephritis in the 4-MU–treated group (Table 1 taken from ref. 3 with permission) (Figure 1 taken from ref. 3 with permission).

**Table 1:** Histopathology and immunohistochemistry findings in kidneys from 4-methylumbelliferone–treated and control mice

Findings	4-MU	Control	*P* Value
**Glomeruli**			
Mesangial expansion score	0.94±0.21 (∼24% of glomeruli affected)	1.24±0.16 (∼31% of glomeruli affected)	0.017
Glomerular injury index	1.19±0.29 (∼30% of glomerular tuft area affected)	1.53±0.23 (∼38% of glomerular tuft area affected)	0.047
% of segmental glomerulosclerosis	9.0%±1.8%	14.9%±7.5%	0.090
% of nodular glomerulosclerosis (Kimmelstiel-Wilson lesion)	3.0% (0.0–13.0)	1.0% (0.0–8.0)	0.87
% of global glomerulosclerosis	6.2% (5.9–10.2)	7.6% (2.6–20.0)	0.76
% of affected glomeruli	54.4%±15.1%	64.6%±10.0%	0.19
% of mesangiolysis	5.6%±2.4%	7.7%±4.9%	0.41
Glomerular diameter (*µ*m)	92.8±5.5	96.2±5.7	0.33
**Vascular/interstitium**			
Severe AH	Not observed	42.9% (3/7)	0.20
% of interstitial fibrosis and tubular atrophy	6.7%±3.9%	6.3%±3.5%	0.85
**Inflammation**			
Nephritis and tubulitis	Not observed	28.6% (2/7)	0.47
CD68^+^ cell count–glomeruli	1.0 (0.5–4.1)	7.6 (2.4–21.0)	0.081
CD68^+^ cell count–interstitium	2.0 (1.1–6.3)	3.8 (1.1–74.2)	0.71
CD44^+^ cell count–glomeruli	27.0 (19.9–46.4)	34.1 (22.3–61.0)	0.57
CD44^+^ cell count–interstitium	100.0 (74.6–115.9)	138.5 (100.5–254.8)	0.18

Adapted from ref. 3 with permission. 4-MU, 4-methylumbelliferone, AH, arteriolar hyalinosis.

Our study suggests that HA accumulation in the kidneys and arterioles is directly involved in the progression of DKD. In addition, 4-MU treatment slowed the progression of DKD in a mouse experimental model by reducing HA accumulation in the kidney and arterioles. This study was the first to show that HA synthesis inhibition led to slowing DKD progression and highlighted, for the first time, that HA synthesis inhibition can be a potential therapeutic target when treating DKD.

## HA Synthesis Inhibitor in Other Diseases

4-MU has been studied as a promising therapeutic agent to prevent metastases of different types of malignant tumor cells in vitro and in animal models and to treat certain autoimmunologic disorders.^39,58^ Results of these studies have been remarkable and exceptionally interesting. These animal experiments have led to phase 2 studies in humans currently listed on ClinicalTrials.gov (accessed January 19, 2023). The first study is a phase 2 study to investigate the use of 4-MU as treatment option for chronic hepatitis B virus and hepatitis C virus (ClinicalTrials.gov Identifier: NCT00225537). The second listed study has no selected phase, but its purpose is to investigate the safety and efficacy of hymecromone (4-MU) tablets for the treatment of patients with coronavirus disease 2019 (COVID-19) infection (ClinicalTrials.gov Identifier: NCT05386420). The third listed study is a phase 2 study exploring the use of oral hymecromone to treat adolescents and adults with primary sclerosing cholangitis (ClinicalTrials.gov Identifier: NCT05295680). And the fourth listed study is a phase 2 study to investigate the use of hymecromone to treat adults with pulmonary hypertension, including interstitial lung disease (The Investigation of H01 in adults With Pulmonary Hypertension Including Interstitial Lung Disease Study) (ClinicalTrials.gov Identifier: NCT05128929). It is important to mention that another listed study is a phase 1 study of oral hymecromone and HA synthesis in healthy volunteers that has been completed and will be discussed more in detail below (ClinicalTrials.gov Identifier: NCT02780752).

## HA Synthesis Inhibitor Use in Humans

Rosser *et al.* have conducted an open-label, single-center, dose-response study of hymecromone (4-MU) in healthy volunteer adults.^59^ Patients received one of the three 4-MU doses of 400, 800, or 1200 mg three times daily to a total of 1200, 2400, or 3600 mg daily. The aim of the study was to assess the safety and tolerability of 4-MU. These researchers found that 4-MU is well tolerated at these tested doses. Serum and sputum 4-MU concentration increased in a dose-dependent manner. There was a significant sputum HA level decrease after a 4-day treatment with 4-MU. The serum HA level also decreased with 4-MU treatment.

Recently, Yang *et al.* published a study on using 4-MU to treat COVID-19 infection and disease progression.^60^ This study was conducted on human patients on the basis of preliminary data showing that severe acute respiratory syndrome coronavirus 2 promotes the COVID-19 progression by upregulating hyaluronic acid. Interestingly, when the study team delivered HA to the lungs of male mice, it resulted in the formation of consolidation and ground-glass opacities similar to what is seen among patients with COVID-19–related lung injury. The research team discovered that HA was closely relevant to clinical parameters, such as lymphocytes, C-reactive protein, D-dimer, fibrinogen, the mass and volume of pulmonary ground-glass opacity, and the mass and volume of consolidation in patients with low HA levels. Consequently, 94 patients with confirmed COVID-19 were treated with 4-MU (doses of 400 mg three times daily for 35 days) until COVID-19 infection resolved. In addition, 50 patients with confirmed COVID-19 infection were enrolled into the control group. The results of this trial showed that treatment with 4-MU resulted in significantly more improvement and resorption in pulmonary lesions as compared with the control group. In addition, 4-MU–treated patients had more improvement in clinical parameters of COVID-19. Importantly, there were no adverse reactions observed in this clinical trial. No difference was seen in liver function tests or kidney function tests between the two groups.

There have been many more clinical studies in humans over the past 4 decades. Nagy *et al.* summarized in their manuscript the clinical studies of 4-MU in humans published up until the time of their manuscript.^39^ We have adapted and expanded their table and added the newly published in-human studies since their publication (Table 2).

**Table 2:** Clinical trials using hymecromone (4-methylumbelliferone) in humans

Reference	Patient Type	Indication	Study Type	*n*	Dose	Primary and Secondary Outcome	Duration	Adverse Events
Walter and Seidel^61^	Patients requiring cholecystectomy, age older than 14 yr	Postsurgical revision of the biliary pathways	Double-blind, randomized, placebo-controlled	25	2400 mg/d×7.5 d, then 1200 mg×7 d	Postoperative gall bladder volume, residual pressure, and serum enzymes	2 wk	Decreased drain output and need for postoperative, two patients with mild headaches in the treatment group, three with decreased appetite and diarrhea in the placebo group
Camarri and Marchettini^62^	After cholecystectomy dyspepsia, age >16, mean 58.5 yr	Treatment of symptoms after surgery on bile ducts	Double-blind, randomized, placebo-controlled	13	800 mg twice daily	Pain and gastroenteric symptoms	3 wk	No unexpected symptoms, no abnormal laboratory results (CBC, Cr, BUN, AST, ALT, Alk phos, glucose, UA) by the end of the treatment
Trabucchi *et al.*^63^	Biliary dyskinesia	Biliary dyskinesia	RCT versus tiropramide 300 mg	20	1200 mg daily	Biliary pain attacks Dyspepsia symptoms	3 mo	NA
Quaranta *et al.*^64^	After cholecystectomy dyspepsia (motor disorders of the biliary tract), age >16, mean 59.5 yr (62 in the active drug group versus 56 in the placebo group)	Treatment of abdominal pain and gastroenteric symptoms because of motor disorders of the biliary tract after cholecystectomy	Placebo-controlled, double-blind, randomized	15	1200 mg/d	Abdominal pain and gastroenteric symptoms	3 wk	One patient developed renal colic/oliguria resolved after drug cessation. No other side effects reported including normal laboratory results after therapy (CBC, uric acid, glucose, UA, AST, ALT, bilirubin, alk phos, *γ*-GT, cholesterol, SPEP, and amylase)
Garretta and Venitz^65^	Healthy, age 21–35 yr	Pharmacokinetics	Pharmacokinetics	8	400 mg IV, 800 mg IV, 600 mg by mouth solution, 1200 mg by mouth solution, 1200 mg tablets	Pharmacokinetics	Once	NA
Krawzak *et al.*^66^	Healthy, age 22–30 yr	Common bile duct contraction after meal	Prospective, double-blind, randomized crossover study	20	400 mg IV	Common bile duct width while fasting and after meal	Once, after meal	NA
Abate *et al.*^67^	Biliary dyskinesia	Dyspepsia Biliary dyskinesia Cholelithiasis Hepatopathy	Placebo-controlled, multicenter, randomized	61	600 mg with lunch, 600 mg with dinner	Abdominal pain	2 wk	NA
Hoffmann *et al.*^68^	Healthy, age 25–37 yr	Healthy	4-MU by mouth and IV	20	800 mg ×1 (by mouth and IV)	Gall bladder volume Common bile duct diameter	Once, with meal	NA
Nersesov *et al.*^69^	Adults, age 18–65 yr	Primary functional disorders of the gallbladder or sphincter of oddi, after cholecystecomy syndrome	Multicenter, prospective, observational	877	Group A (*n*=89) 600 mg/d Group B (*n*=788) 1200 mg/d	Biliary pain severity on the basis of a ten-point visual analog scale	21 d	Treatment satisfaction was higher in group B
Rosser *et al.*^59^	Healthy adults, age 18–65 yr	Phase 1 Healthy adult volunteers	Dose response—phase 1 Open-label, nonrandomized	12	400 mg ×3, 800 mg ×3, or 1200 mg ×3	Safety Tolerability Dose response	4 d	Excellent safety and tolerability
Yang *et al.*^60^	Patients with COVID-19	COVID-19	Open-label random trial to assess clinical parameters	94	94 patients received 400 mg ×3 daily and 50 patients' control	Primary: changes in lymphocyte counts, CRP, fibrinogen, D-dimer Secondary: changes in pulmonary CT	35 d	None

Partially adapted, expanded, and updated from ref. 39 with permission. 4-MU, 4-methylumbelliferone; CBC, complete blood count; AST, aspartate aminotransferase; ALT, alanine aminotransferase; COVID-19, coronavirus disease 2019; CRP, C-reactive protein; CT, computed tomography; GT, gamma-glutamyl transferase; UA, urinalysis; SPEP, serum protein electrophoresis; IV, intravenous; NA, not applicable.

## Global Availability of 4-MU at Present

4-MU is available under other names outside the United States, such as hymecromone, and has been approved in Europe for human use for biliary dyskinesia since July 27, 1960.^39^ 4-MU has no anticoagulation properties although it is a coumarin derivative. Several human trials have been published, and all have shown excellent safety and tolerability profile (Table 2, which was adapted from reference 39 and expanded). The limitation with these trials is their duration as the longest trial was for only 3 months and was for the indication of biliary dyskinesia. The approved dose in Europe is 300–800 mg three times daily by mouth (which is 900–2400 mg daily). The most common side effects from these trials were diarrhea (1%–10%, dose dependent) and other mild gastrointestinal symptoms. Contraindications to taking 4-MU include pregnancy and lactation because of the lack of safety data available for these two groups.^39^

## Future Directions for HAI in DKD

AH and increased HA deposit in kidneys, seen among patients with DM and DKD, have been shown to be associated with increased ESKD incidence, worse proteinuria, increased cardiovascular events, worse renal graft survival, and worse glomerular disease outcomes.^42,46^ Our study has shown the benefits of 4-MU, a HA synthesis inhibitor, in slowing DKD progression in animal experimental models with potential important benefit in glucose control.^3^

Phase 1 study on 4-MU among healthy human adults has been completed and published, highlighting its safety and tolerabiltiy.^59^ On the basis of all of this in addition to the results of other in-human published studies, we believe that it is time for a phase 2 in-human study to evaluate the safety and efficacy of using 4-MU among patients with DKD. Such study will, if supported by findings, provide valuable data for implementation of a phase 3 clinical trial.
